# Human papillomavirus typing of invasive cervical cancers in Italy

**DOI:** 10.1186/1750-9378-1-9

**Published:** 2006-12-27

**Authors:** Annarosa Del Mistro, Helena Frayle Salamanca, Rossana Trevisan, Roberta Bertorelle, Anna Parenti, Emanuela Bonoldi, Paola Zambon, Daria Minucci

**Affiliations:** 1 Immunologia e Diagnostica Molecolare Oncologica, Istituto Oncologico Veneto, Padova, Italy; 2 Anatomia Patologica, Azienda Ospedaliera di Padova, Padova, Italy; 3 Anatomia Patologica, Ospedale di Vicenza, Vicenza, Italy; 4 Registro Tumori Veneto, Istituto Oncologico Veneto, Padova, Italy; 5 Ostetricia e Ginecologia, Azienda Ospedaliera di Padova, Padova, Italy

## Abstract

**Background:**

Human papilloma viruses (HPV) are the necessary cause of invasive cervical cancer (ICC). Of the many different types identified so far, only a few of them account for the great majority of cases worldwide, with geographical differences in their distribution. Data on the local distribution are now of interest in view of the soon-to-come introduction of HPV type-specific prophylactic vaccines.

**Results:**

We have investigated HPV type distribution in samples of 48 ICC cases occurred in women living in North-East Italy in the years 1997–1999. Cases were extracted from the Venetian Tumour Registry files, as incident cases whose specimens had been processed in two Pathology Departments. Search and typing were performed by polymerase chain reaction (PCR) using GP5+/GP6+ primers, followed by direct sequencing or reverse dot blot. Three cases were PCR negative using the housekeeping primers and hence excluded. One case was negative by all HPV tests used. HPV 16 was present in 32 (72.7%) cases, as single infection in 28, in mixed infection in 4. Of the 44 positive cases, HPV 16 and HPV 18 accounted for 33 (75%), as single or mixed infections. The other high risk HPV types accounted for 11 (25%) of the remaining infections. Of the 32 HPV 16 positive cases, sequencing of the E6 gene could be performed in 25; the prototype isolate was identified in 7, and the variant T350G in 18; in 4 cases one or more additional mutations were present.

**Conclusions:**

Our results suggest that HPV 16 has a very high prevalence among women with invasive cervical cancer in Italy; therefore, the use of a prophylactic vaccine for HPV types 16 and 18 could prevent up to 75% of invasive cervical cancers in Italy.

## Findings

Persistent infection with high risk HPV is a necessary cause for invasive cervical cancer; studies on HPV type distribution among ICC cases worldwide have shown some geographical variation [1]. Limited data on HPV type distribution among ICC cases from Italy are available to date [2]; this information is useful in view of the availability of type-restricted prophylactic vaccines in the very near future. We retrieved consecutive incident cases of ICC occurred during the years 1997–1999 in Italian women living in the North-East area from the Venetian Tumour Registry files, and selected the cases whose samples had been processed in the Pathology Departments of Padova and Vicenza.

A total of 48 ICC cases were selected; 43 squamous cell cancers (SCC) and 5 adenocarcinomas. Median age at diagnosis was 55 yrs (range 28–89). DNA was extracted from formalin-fixed paraffin-embedded samples by using the QIAamp DNA minikit (Qiagen GmbH, Germany), according to the manufacturer's instructions. To verify DNA quality, amplification of a 268 bp fragment of the Beta-globin gene was performed using PC04/GH20 primers. HPV detection was conducted using GP5+/GP6+ general consensus primers (which amplify a fragment of approximately 140–150 bp), as previously described [3], and 0.5 U of AB SuperTaq (AB Analitica, Padova, Italy). HPV types were identified by direct sequencing; PCR products were purified by ExoSAP-IT (USB Corporation, Ohio, USA) and subjected to cycle sequencing by ABI PRISM Big Dye Terminator Cycle Sequencing kit (Applera, Foster City, CA, USA), and sequencing reactions were run on the ABI PRISM 310 Genetic Analyzer (Applied Biosystems). The BLAST server was used to match all sequences available in GenBank [4]. HPV 16 type specific primers H16L1/H16R3 were used to amplify a 323 bp fragment in the E6 gene, as previously described [5], and the fragments subjected to direct sequencing (from both directions) to characterize the isolates; variants were defined by comparison to HPV 16 prototype sequences. HPV 16 and HPV 18 type specific primers amplifying fragments of 98 bp and 118 bp, respectively, were also used, as previously described [6]. Samples resulted negative for HPV DNA or untypable by direct sequencing and type-specific PCRs were further tested by nested PCR and reverse dot blot by using the HPV-HS BIO and HPV Strip Detection kits (AB Analitica, Padova, Italy), according to the manufacturer's instructions, which allow the detection of HPV types 6, 11, 34, 40, 43, 44, 53, 54, 61, 69, 70, 16, 18, 31, 33, 35, 39, 45, 51, 52, 56, 58, 59, 66.

Of the 48 cases analyzed, 3 SCC resulted negative for the housekeeping gene and were excluded, and 1 SCC was negative for HPV DNA with all the tests used. The remaining 44 cases (97.8%) were positive for HPV sequences; 39 single infections and 5 (11.4%) double infections, as described in Table 1. In 8 cases the type could be determined only by nested PCR followed by reverse dot blot, and all the 5 mixed infections were in this group. Overall, the most frequently detected types were HPV 16, 18 and 33 (cumulatively, 37/44, 84.1%). HPV 16 E6 gene could be sequenced in 25 cases; in 6 cases amplification with the E6 type specific primers was negative, and in 1 the amplified fragment could not be successfully sequenced. The E6 pattern corresponded to the prototype in 7 cases, while the T350G mutation was present in 18; as single mutation in 14, with another mutation in 3 (T256C, A257G, A442C, respectively), and with three additional mutations in 1 (T286A, A289G, C335T).

**Table 1:** HPV type distribution in relation to histology among 44 HPV positive invasive cervical cancers.

HPV types	Histology	
	
	Squamous cell carcinoma	Adenocarcinoma
HPV 16	26	2
HPV 16 + HPV 18	3	
HPV 16 + HPV 31	1	
HPV 18		1
HPV 33	2	2
HPV 35	1	
HPV 45	2	
HPV 52	1	
HPV 56 + HPV 70	1	
HPV 58	2	

Overall, 44 out of the 45 valuable ICC Italian cases occurred during 1997–1999 were HPV positive. HPV 16 was detected in 32 cases (72.7%), a result indicating a very high prevalence in Italy, in keeping with other studies [2,7,8]. Indeed, in our region HPV 16 is the most prevalent type also in high grade lesions; 40% of samples cytologically diagnosed as high grade squamous intraepithelial lesions [9], and 65.5% (36/55) of histologically confirmed CIN3/CIS (data not shown). These data are in line with the notion that HPV 16 positive infections are at great risk of clinical progression and need a closer follow-up, as recommended also by other authors [10]. This implies that when searching for HPV sequences in clinical samples, careful attention must be paid to the detection of type 16; in our experience, testing by HPV 16 type-specific PCR in adjunct to consensus primers PCR is very useful to guarantee high sensitivity for this type, since HPV 16 infections can sometimes be missed by consensus primers in cases of mixed infections and/or low viral load. A mixed infection was detected in 5 (11.4%) of our cases; this result is somehow surprising, but mixed infections have been detected in invasive cervical cancers also by other authors [11]. Indeed, different data have been obtained so far on the frequency and the role in cervical neoplasia development of coinfections with multiple HPV types. This can be due to differences in the studied population or in the typing methodology used. In our study the five double infections were all found by nested PCR followed by reverse dot blot, and it is known that reverse hybridization methods are more sensitive than sequencing in detecting multiple genotypes [12].

Sequence analysis of the E6 gene in 25 HPV 16 positive ICC cases showed the presence of the T350G mutation in the great majority (18/25, 72%), in 4 cases also accompanied by other mutations; among the mutations identified, A257G, C335T, T350G, A442C lead to amino acid changes (I52V, H78Y, L83V, E113D, respectively), while C256T, T286A, A289G are silent not leading to amino acid changes [13,14]. Interestingly, the A257G mutation has been firstly described very recently, and occurred in combination with the T350G variant [14], as in our case. Variations in the viral genes leading to amino acid substitutions can alter the biological functions of the encoded proteins or their antigenic properties. Of the naturally occurring HPV 16 viral variants, the T350G (L83V) is the most commonly found among invasive cancers [15], and has been linked to an increased risk for cervical disease progression [14].

HPV 33 was detected in 4 cases, resulting the second most frequent type (together with type 18), a result observed also in The Netherlands [16].

In conclusion, our results suggest that HPV 16 has a very high prevalence among women with invasive cervical cancer living in Italy, implying that a prophylactic vaccine for types 16 and 18 could prevent up to 75% of cancer cases in Italy.

## Abbreviations

HPV (Human papilloma viruses); ICC (invasive cervical cancer); SCC (squamous cell carcinoma); CIN3 (cervical intraepithelial neoplasia); CIS (carcinoma in situ); PCR (polymerase chain reaction).

## Authors' contributions

ADM planned and coordinated the study, analyzed the data and drafted the manuscript. HFS and RT performed the molecular analyses. RB participated in the sequence alignment and helped to draft the manuscript. AP, EB, PZ and DM participated in the design and coordination of the study, and in the collection, assembly and review of the patients' data. All authors read and approved the final manuscript.
